# The International Breakfast Research Initiative—Evaluation and Comparison of Breakfast Nutrient Intakes in Indonesia, Malaysia and the Philippines with a View to Proposing a Harmonised Nutrient-Based Breakfast Recommendation

**DOI:** 10.3390/nu16142180

**Published:** 2024-07-09

**Authors:** Michael J. Gibney, Imelda Angeles-Agdeppa, Elise Line Mognard, Helda Khusan, Jean-Pierre Poulain, Apple Ducay, Marvin B. Toledo, Roselynne Anggraini, Judhiastuty Februhartanty, Sinéad Hopkins

**Affiliations:** 1 Institute of Food and Health, University College Dublin, Dublin D04 V1W8, Ireland; 2 Department of Science and Technology, Food and Nutrition Research Institute, Bicutan, Taguig 1631, Philippines; iangelesagdeppa@yahoo.com.ph (I.A.-A.);; 3 Chair “Food Studies: Food, Cultures & Health”, Taylor’s University, Subang Jaya 47500, Malaysia; eliseline.mognard@taylors.edu.my (E.L.M.); or poulain@univ-tlse2.fr (J.-P.P.); 4 Centre for Asian Modernisation Studies (CAMS), Taylor’s University, Subang Jaya 47500, Malaysia; 5 Faculty of Social Sciences and Leisure Management, Taylor’s University, Subang Jaya 47500, Malaysia; 6 Centre d’Études et de Recherche: Travail, Organisation, Pouvoir (CERTOP) UMR CNRS 5044, Université de Toulouse, 31058 Toulouse, CEDEX 9, France; 7 Faculty of Health Sciences, Universitas Muhammadiyah Prof Dr HAMKA, Jakarta 12130, Indonesia; helda_khusun@uhamka.ac.id; 8 Southeast Asian Ministers of Education Organization Regional Centre for Food and Nutrition (SEAMEO RECFON)—Pusat Kajian Gizi Regional (PKGR), Universitas Indonesia, Jakarta 13120, Indonesia; lynne.prigel@gmail.com (R.A.); jfebruhartanty@seameo-recfon.org (J.F.); 9 Nutrition Department, Faculty of Medicine, Universitas Indonesia—Dr. Cipto Mangunkusumo General Hospital, Jakarta 10430, Indonesia; 10 Department of Nutrition, Faculty of Sports and Health Sciences, Universitas Negeri Surabaya, Surabaya 60231, Indonesia; 11 Cereal Partners Worldwide S.A., 1000 Lausanne, Switzerland

**Keywords:** breakfast, nutrient recommendations, Indonesia, Malaysia, Philippines, IBRI

## Abstract

The International Breakfast Research Initiative is a global study of breakfast nutrition, involving 17 countries in four continents, aiming to derive nutrient-based regional recommendations for breakfast. This study aimed to propose a harmonised recommendation for three South-East Asian countries: Indonesia, Malaysia, and the Philippines. For each country, data from nationally representative dietary surveys on the contribution of breakfast to daily nutrient intakes at both the adult population level and at the level of the upper tertile of daily nutrient density using the Nutrient Rich Food (NRF) Index were collated and examined. Energy intakes at breakfast ranged from 26 to 27% of daily energy intake. In all three countries, breakfast was carbohydrate-rich, providing 52 to 72% of breakfast energy intake, while it was higher in total and saturated fat in Malaysia and Indonesia. Intakes of fibre and vitamin C were low in all countries, while Malaysia tended to have higher intakes of most minerals, including sodium. Daily and breakfast nutrient intakes (at the population level and in the upper tertile of the NRF Index) were compared to the Codex Alimentarius nutrient reference values (NRVs) to assess adequacy. A decision tree was established based on these data to guide the development of recommendations for nutrient intakes at breakfast across the three countries.

## 1. Introduction

The International Breakfast Research Initiative (IBRI) was established as a global project to examine the contribution of breakfast to total daily nutrient intake in a selection of representative countries for a geographical region and to use the data to explore the development of harmonised nutrient guidelines for breakfast. Existing guidelines for breakfast are most commonly issued at the food-based level, with only a few available at the nutrient level. An example of the former is the fact sheet on breakfast issued by the British Dietetic Association [1]. In the case of nutrient recommendations for breakfast, the US National Academies of Science, Engineering and Medicine issued a guideline for school breakfasts of 21.5% for micronutrients based on the mid-point range (19–24%) observed in the School Nutrition Dietary Assessment Study III [2,3]. The methodology of the IBRI project differed in that it set out to establish the contribution of breakfast to daily nutrient intake, not only at the average or median population level, but also in the top tertile of the population distribution of daily nutrient density as measured using the NRF Index [4], and to use these data to serve as a basis for proposing nutrient recommendations.

The derivation of quantitative recommendations for nutrient intake at breakfast is important as it can assist policy makers and healthcare professionals to underpin and improve the existing food-based guidelines and standards on breakfast. These guidelines, for example, can play a role in guiding the provision of breakfast in public institutions like schools. Based on these nutrient recommendations, food-based culturally appropriate guidelines can be developed to aid education as regards food choice at breakfast. Moreover, they could assist policy makers and industry in the process of re-formulation of breakfast products to optimise their nutrient profiles in the context of the breakfast meal.

Across the globe, food choices, food habits and eating traditions vary such that a universal set of dietary guidelines for breakfast is neither possible nor desirable. Ethnic, religious, and socio-cultural factors play a major role in this variability. In the current study, we sought to expand the IBRI project to the South-East Asian region, where breakfast habits are known to differ markedly from other global regions studied in the IBRI project (Europe/North America [5] and Latin America [6]). The three countries included were Indonesia, Malaysia, and the Philippines [7,8,9], and their selection was based on the availability of up-to-date and nationally representative dietary data on breakfast consumption habits. In all three countries, breakfast is promoted as a health-promoting behaviour. However, only Indonesia’s Dietary Guidelines [10] provide specific nutritional targets for breakfast, recommending that it should broadly account for 15–30% of daily nutrient needs. In the Philippines, the Food and Nutrition Research Institute provide guidance for what constitutes a nutritionally adequate one-day menu. For breakfast, it is advised that adults eat 1–1.5 bowls of rice, a fried egg and fish accompanied by a piece of fruit like papaya and a powdered milk drink [11].

The first phase of the IBRI methodology is to conduct a detailed assessment of nutrient intakes at breakfast and the contribution of breakfast towards daily nutrient intakes in each country. This phase has been completed and national-level findings have been published for Indonesia, Malaysia, and the Philippines [7,8,9]. The second phase involves evaluation and consolidation of the results across countries and application of the IBRI methodology to propose a harmonised nutrient-based breakfast recommendation. Therefore, the objectives of the present study were, firstly, to evaluate and compare breakfast nutrient intakes across the three countries, drawing on published findings, and, secondly, to utilise these data to propose a harmonised nutrient-based recommendation for the breakfast meal. It is anticipated that the proposed recommendations can serve as a guide for other countries in the region that have similar dietary patterns but lack the resources to conduct detailed dietary analyses of their populations.

## 2. Materials and Methods

### 2.1. Dietary Survey Methodology

Full details of the national dietary intake surveys used to extract data on breakfast and daily nutrient intakes in the three countries are available in published papers [12,13,14]. The Malaysian and Indonesian dietary data are from secondary analyses of the Malaysian and Indonesian Food Barometer studies, respectively [12,13]. These are cross-sectional dietary and socio-anthropological surveys based on the same methodology. In Malaysia, a sample of 1608 adults aged 18 years and over were recruited based on random complex multistage sampling with the aim of ensuring their representativeness in terms of geographical regions within Peninsular Malaysia (Sabah and Sarawak), degree of urbanisation, age, and ethnicity. The sampling stratification methodology was the same as for the first Malaysian Food Barometer study conducted in 2013 (*n* = 2000), which was based on the 2002–2003 Malaysian Adult Nutrition Survey (MANS, *n* = 6928) conducted by the Ministry of Health. The Indonesian Food Barometer applied a similar multistage random sampling procedure and resulted in a sample size of 1665 adults aged 18 years and over. To ensure representativeness, the survey was conducted in six provinces of Indonesia, where 48% of the population resides (West Sumatra, Jakarta, West Java, East Java, Bali, and South Sulawesi). Furthermore, the survey sample was weighted against the Indonesian Census Data.

Data for the Philippines came from the 2018 Expanded National Nutrition Survey (ENNS), where a two-stage sampling technique was utilised [14]. In the first stage, Primary Sampling Units (PSUs) were selected, representing sampling domains from 81 provinces. Each domain had 16 sample replicates drawn from it. The second stage involved selecting households from these 16 sample replicates, with the selected households serving as the final sampling unit. The final sample size consisted of 63,655 individuals aged 6 years and above. Only the dietary data for adults aged 19–59 years (*n* = 32,255) were used for the present analysis. All adult samples used for the current study excluded pregnant and breastfeeding women at the time of recruitment.

### 2.2. Dietary Assessment

Single face-to-face 24 h recalls were used to collect dietary data in Malaysia and Indonesia. The interview followed a structured questionnaire to capture all foods (including recipes) and beverages consumed in the previous 24 h, applying the multiple-pass method. For each food occasion on the day of the interview, the participants were asked to name the eating occasions themselves. Food photographs were used to estimate portion size, and recalls were conducted over both weekdays and weekends. In the Philippines, two-day non-consecutive 24 h recalls were conducted face to face with the participants. The first 24 h recall was collected for all participants, while the second was completed in 50% of randomly selected households to estimate the day-to-day variation in nutrient intakes.

In Malaysia, energy and nutrient intakes were estimated using NutritionistPro version 8.1, which included the data of the Malaysian Food Composition Table (MyFCD), updated in 2017. In Indonesia, a customised version of the NutriSurvey for Windows 2007 software was used to estimate energy and nutrient intakes. The Indonesian Food Composition Tables (FCTs) 2017 were primarily utilised, but the Philippines’ FCTs 2019 were employed for estimation of total sugar, saturated fatty acids, magnesium, vitamin E and vitamin D. In the Philippines, estimated energy and nutrient intakes were processed using the electronic-based Individual Dietary Evaluation System (IDES) developed by the Food and Nutrition Research Institute, which contains the data of the updated Filipino FCTs. In Indonesia, under- and overestimation of energy intake was estimated using the Goldberg criteria and resulted in the exclusion of 332 participants, with a remaining sample size of 1333. In Malaysia, 4 participants with implausible energy intakes (defined as below 500 kcal) were excluded from the dataset, resulting in a final sample size of 1604.

### 2.3. Definition of Breakfast

In both Malaysia and Indonesia, breakfast was defined as the “first meaningful” intake of food and/or drink consumed before 10:00 a.m. containing at least 50 kcal and identified by the respondent as breakfast or its equivalent in the local language. If there was more than one food intake occasion before 10:00 a.m., the food occasion selected as breakfast was the food occasion defined by the respondent as breakfast or morning snack or as the food occasion having the highest energy content. Breakfast skippers were defined as participants who reported no intake before 10:00 a.m. or had an intake of <50 kcal before 10:00 a.m. In the Philippines survey, breakfast was defined based on the meal code number 1 indicated in the questionnaire and a self-report of “breakfast” by the interviewee. Breakfast skipping was defined as no breakfast consumption, or less than 50 kcals of energy consumed at breakfast.

### 2.4. Assessment of Diet Quality

As indicated, the focus of the IBRI project is not only on average or median nutrient intakes at breakfast and at daily level, but also on the pattern of nutrient intake of those within each population in the top tertile of the NRF Index [4]. The NRF Index version 9.3 is based on the daily intake of nine nutrients to encourage (protein, fibre, vitamin A, vitamin C, vitamin D, calcium, iron, magnesium, and potassium) and three nutrients to limit (saturated fat, total or added sugar, and sodium). None of the three countries had data on added sugars, so total sugar intake was used instead. The index expresses intake as a % of a reference dietary value (DV) or a maximum reference value (MRV) using the following equations:

$$NRF\;9.3=\left(NR-LIM\right)\times 100\;NR=\sum_{i=1}^{9}\frac{\ln take_{i}/Energy\times 2000}{DV_{i}}\;LIM=\sum_{i=1}^{3}\frac{\ln take_{i}/Energy\times 2000}{MRV_{i}}$$

### 2.5. Use of Reference Values

In both Malaysia [15] and the Philippines [16], the national dietary reference values were used in the assessment of the NRF 9.3 Index, while in Indonesia, national reference values for nutritional labelling were used. These labelling values are a consolidated version of the age- and sex-specific national dietary reference values [17]. In the present study, the collated nutrient intake data across the three countries were expressed as a percentage %s of the Codex NRVs [18] for nutritional labelling to create a standardised base to assess the data and on which to propose quantitative nutrient intake guidelines for the three countries combined.

### 2.6. Statistical Analysis

Statistical analyses were performed using SPSS versions 26 and 20 (IMB, New York, NY, USA) in Malaysia and Indonesia, respectively, and in the Philippines STATA version 15 (StataCorp, College Station, TX, USA) software was used. Means and standard deviations for daily and breakfast energy and nutrient intakes were calculated in Malaysia, while in Indonesia medians and inter-quartile ranges were calculated due to skewness in the data. In the Philippines, PCSIDE version 1.02 (Iowa State University, Ames, IA, USA) was used to estimate the usual mean nutrient intakes with standard errors. Continuous variables, including breakfast nutrient intakes, were compared across tertiles of NRF scores using Analysis of Covariance in Malaysia and Indonesia and using a trend test in the Philippines. The significance level was set as *p* < 0.05.

### 2.7. Guiding Principles for the Development of Nutrient Recommendations for Breakfast

The guiding principles for developing the nutrient recommendations followed a similar approach to previous IBRI studies, but some adaptation was needed.

With respect to public health-sensitive nutrients, including saturated fat, free sugars, and sodium [19,20,21], and also carbohydrates [22], the relevant guidelines of the WHO were used as reference values. For protein, fibre and micronutrients, the starting point was an evaluation of the average daily intake relative to the Codex NRVs. If these intakes were close to the NRVs, it was agreed that prevailing intakes at breakfast need not be altered and could be set as the target. Where the average daily nutrient intake fell short of the Codex NRVs, then some adjustment upwards was needed and data on the contribution of daily breakfast intake were used to indicate if breakfast was a target meal to reduce this deficit. Several options were considered for each nutrient: the average intakes at breakfast, the average intakes at breakfast in the upper tertile of the NRF 9.3 Index and the use of a default value of 20% of daily intake, which is the minimum energy target for breakfast based on observed intakes. The exact extent to which these prevailing intakes needed to be adjusted upward was based on the partners’ judgement and pragmatism.

## 3. Results

### 3.1. Socio-Demographics of Breakfast Consumers

Table 1 outlines the socio-demographic characteristics of breakfast consumers in each of the three countries. Of the initial samples of 1604, 1333 and 32,255 participants in Malaysia, Indonesia, and the Philippines, 89%, 95% and 96% were classified as breakfast consumers, respectively. Adults aged 30–59 years made up the majority of the breakfast-consuming sample in each country. There were roughly 50% females and 50% males in each sample, with the majority living in urban areas, especially within Malaysia (91%). Education levels were highest in Malaysia, with 90% of the participants having a secondary-level education or higher compared to 73% and 54% in the Philippines and Indonesia, respectively. In each country, just over 50% of each sample were in the normal weight range, while around 35% were either overweight or obese and 7–10% were underweight. In Malaysia, 54% of the sample were Malays, 25% were Chinese, 14% were non-Malay Bumpiutra and 7% were Indian [8]. Further characterisation of the samples by socio-demographics and breakfast consumption groups can be found in each country’s publication.

### 3.2. Breakfast Nutrient Intakes of Breakfast Consumers across the Three Countries

Table 2 outlines the intake of nutrients at breakfast for all breakfast consumers across the three countries. The mean breakfast energy intakes in Malaysia and the Philippines were 474 kcal and 426 kcal, respectively, while the median intake for Indonesia was 396 kcal. Total carbohydrate intake at breakfast was notably higher in the Philippines (76.9 g) than in Malaysia (62 g) and Indonesia (50.9 g), while total fat and saturated fat followed the opposite pattern, being higher in Malaysia and Indonesia than in the Philippines. Total sugar intakes at breakfast were 1.9 g, 10.9 g and 17 g in Indonesia, the Philippines and Malaysia, respectively. Protein intakes at breakfast were comparable across the countries (13–16 g), while fibre intakes were similarly low (1.5–3.2 g) in all countries.

With respect to micronutrient intake at breakfast, all countries had comparable intakes of thiamin, riboflavin, and niacin, while vitamin C intake was very low in all the countries. Malaysia tended to have higher intakes of iron, magnesium, zinc, and potassium than the other two countries. Furthermore, Malaysia had the highest intake of sodium, which was almost double the intakes of the other countries. Calcium intakes were also higher in Malaysia and Indonesia compared to the Philippines. Vitamin A intake was noticeably higher in Indonesia compared to the other countries, and, in contrast, vitamin D intake was notably lower in Indonesia.

### 3.3. Breakfast Nutrient Intakes Relative to Daily Intakes

**Figure 1:**
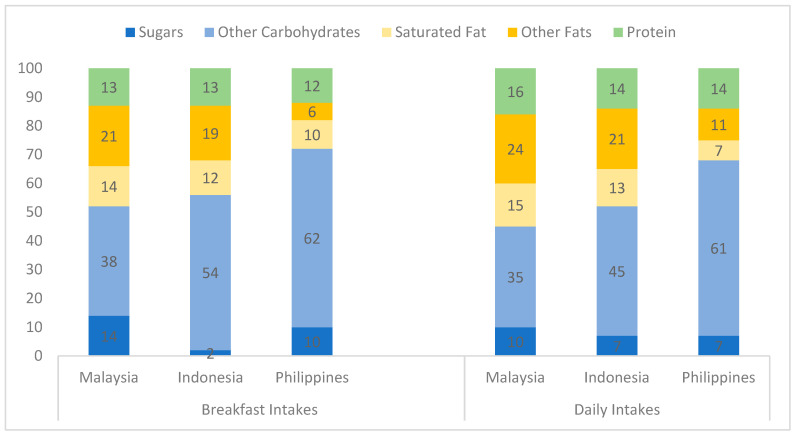
Percentage contributions of macronutrients to energy intakes at breakfast and for the whole day in the three countries.

Figure 1 shows macronutrient intakes expressed as percentages of breakfast energy and daily energy intake across the three countries. Breakfast was typically rich in total carbohydrates, providing around 52–56% of breakfast energy in Malaysia and Indonesia and as much as 72% in the Philippines. However, there were marked differences in the contribution of total sugars to breakfast energy: 2% for Indonesia, 10% for the Philippines and 14% for Malaysia. In the Philippines and Malaysia, the percentage of daily energy from total sugars was lower than that from breakfast (7–10%), while in Indonesia it was higher (7%). Breakfast in Malaysia and Indonesia contributed approximately twice the level of energy from total fat and saturated fat compared to breakfast in the Philippines. Protein contributed 12–13% of the breakfast energy across the three countries and was just slightly lower than the daily contribution (14–16%).

**Figure 2:**
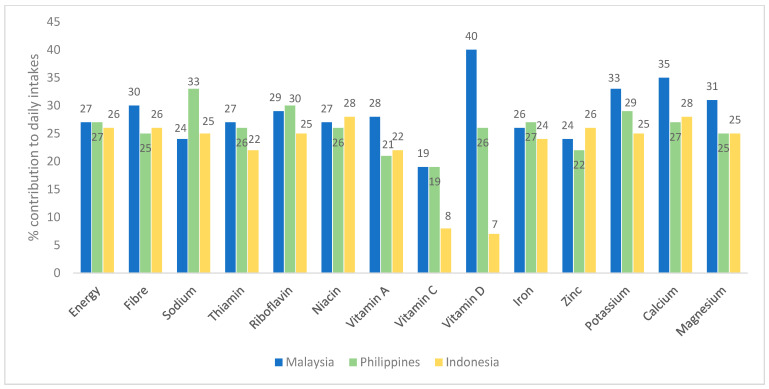
The contribution (%) of breakfast to total daily intake for energy, fibre, and micronutrients in the three countries.

Figure 2 shows the percentage contributions of breakfast to total daily intake for energy, fibre, and micronutrients in the three countries. The contribution of breakfast to energy intake was similar in each country (26–27%). Fibre and most micronutrients showed a % contribution close to the energy contribution level. However, in Malaysia, the contribution of breakfast to daily intakes of fibre, potassium calcium and magnesium (30–35%) exceeded the energy contribution of breakfast (27%). In each country, the contribution of vitamin C from breakfast to daily intakes was lower than that of the energy contribution, and in the case of vitamin D, there was a wide variation across the countries, ranging from 40% of daily intake for the Philippines to 13% for Indonesia.

### 3.4. Daily Nutrient Intakes, Breakfast Intakes and Breakfast Intakes in the Upper Tertile of the NRF 9.3 Index Expressed as a Percentage of Codex NRVs

Table 3 outlines Codex NRVs and nutrient intakes (expressed as %s of Codex NRVs) for the whole day, at breakfast for all breakfast consumers and at breakfast in the upper tertile of the NRF 9.3 Index across the three countries. In terms of total daily intakes (expressed as %s of Codex NRVs), several nutrients were at intake levels that would cause concern. The ranges of daily intakes across the three countries were 25–48% for fibre, 36–64% for vitamin D, 13–84% for vitamin C, 41–63% for calcium, 50–56% for zinc and 30–66% for potassium. For most of these nutrients, intakes at breakfast were generally quite low both for all breakfast consumers and for those in the upper tertile of the NRF 9.3 Index. For example, the fibre breakfast intakes of all breakfast consumers were 13%, 6% and 12% of the Codex NRVs for Malaysia, Indonesia, and the Philippines, respectively, and for those in the upper tertile of the NRF 9.3 Index, intakes were 12%, 8% and 12%. On the other hand, there were some nutrients that showed a trend towards higher intakes in breakfast consumers in the upper tertile of the NRF 9.3 Index, including calcium, iron, magnesium, vitamins A and D, riboflavin, and thiamine. In the case of sodium, the daily value for Malaysia was high at 150% of the Codex NRV, and breakfast contributed approximately 37% to this, while intakes in Indonesia and the Philippines were well within the maximum limit (12–14% of the Codex NRV). Daily protein intakes appeared adequate in all countries, with breakfast contributing 26–32% of the Codex NRV.

### 3.5. Guiding Principles and Proposed Nutrient Recommendations for Breakfast

Table 4 outlines the proposed nutrient recommendations for adults in the three South-East Asian countries. These recommendations were developed and agreed upon by all participating centres following a set of guiding principles outlined below.

Principle 1. This principle governs energy intake, and the recommended value is based on the range of observed intakes of energy from breakfast across the three countries (approximately 400–480 kcal) expressed as a percentage of an average daily energy requirement of 2000 kcal/day. The proposed energy target is therefore 20–25% of the daily energy requirement, or 400–500 kcal.

Principles 2, 3 and 4 govern intakes of protein, carbohydrate, fibre, and those micronutrients for which data were available in the national food composition databases. Given the variability in intakes across the countries and the variability in daily intakes relative to the Codex NRVs, as outlined in Table 2, a single approach for all these nutrients was not possible.

Principle 2. This principle applies to nutrients where the average daily intake of the three populations is close to optimal relative to the Codex NRVs. Since these averaged intakes are presumed to be optimal, the aim is to preserve the nutrient density of prevailing breakfast patterns, and thus there was no need to refer to the intakes in the upper tertile of the NRF 9.3 Index. This principle applies to protein and niacin. In the case of the latter, the average intake across all three countries was 26% of the Codex value. Accordingly, for niacin, a recommended value of 25% of the Codex NRV was chosen. This value is easily attainable in Malaysia and the Philippines, while in Indonesia it is attainable within the top tertile of the NRF 9.3 Index. Principle 2 also applies to total carbohydrates, as breakfast was a carbohydrate-rich meal in all three countries, providing from 52 to 72% of breakfast energy. Therefore, we applied the WHO’s recommendation of a 55–75% energy contribution from carbohydrates [22].

Principle 3. This principle applies to nutrients where the average intakes of the three population groups were low relative to the Codex NRVs. Therefore, it was relevant to consider the intakes in the upper tertile of the NRF 9.3 Index. Thus, in the case of calcium, the overall average intake at breakfast across all three countries was 16% of the Codex NRV. The average intakes of calcium at breakfast for those in the upper tertile of the NRF 9.3 Index was 19%. Accordingly, a recommended value of 20% was proposed. This principle applies to calcium, iron, magnesium, vitamins A and D, riboflavin, and thiamin. In the case of sodium, which is a maximum target, the recommendation was set at a maximum of 15% of the Codex NRV based on the average intakes of sodium in the upper tertile of the NRF 9.3 Index across the three countries.

Principle 4. This principle also applies to nutrients where the average intakes of the three population groups were low relative to the Codex NRVs. However, for these nutrients, the average intakes at breakfast were similarly low for both the total population and for those in the upper tertile of the NRF 9.3 Index. Therefore, a target aspiring to the minimum energy target of 20% was set, also taking into consideration the likelihood of reaching the target. For example, for zinc, the average breakfast intake as a % of the Codex NRV was just 10%, increasing to only 12% when the upper tertile of the NRF 9.3 Index was considered. Thus, it was agreed that the recommended value for zinc at breakfast be set at 15–20% of daily intake. Whilst meeting this value might be a challenge, overall, zinc intakes were sufficiently low to be of public health concern. This principle applies to fibre, vitamin C, potassium, and zinc.

Principle 5. This principle covers macronutrients of public health concern that should be limited, including total fat, saturated fat, and free sugars. As per previous IBRI studies, including studies on Latin America, North America and Europe, the default value was the value recommended by the WHO [19,20,21] applied to breakfast energy intakes.

The final set of recommendations are expressed as %s of Codex NRVs, but they can be adapted and expressed as %s of national age- and gender-specific RDAs.

## 4. Discussion

The present paper represents the final phase of the IBRI project, which aimed to propose an approach to establishing quantitative guidelines for nutrient intake at breakfast on a regional basis. Such guidelines have been developed in phase 1 for Europe and North America and in phase 2 for South America [5,6]. The countries in the current phase were chosen due to the availability of up-to-date, nationally representative dietary intake data. For each country, this is the first time that such comprehensive, nationally representative data on breakfast consumption have been generated, and thus they can empower local investment in public health nutrition programmes to improve nutrient intakes at breakfast.

The results show that breakfast was carbohydrate-rich in all countries, especially in the Philippines, while it was noticeably higher in total and saturated fat in Malaysia and to a somewhat lesser extent in Indonesia. Sodium and total sugar intakes were also notably higher in Malaysia than in the other two countries. These differences are likely explained by the typical foods that are consumed at breakfast in these countries. Although not reported in the current paper, estimates of food intakes at breakfast have been described in published papers for Indonesia [7] and the Philippines [9], while an ethnographic analysis of the Malaysian food intake data is available [21]. In Indonesia and the Philippines, breakfast was characterised by a high intake of rice (approximately 100 g per day at breakfast; >60% consumers). In the Philippines, there was also a high coffee consumption rate (≈60% consumers) but a low rate of consumption of fresh fish and vegetables (<20% consumers). In Indonesia, although vegetable and vegetable products and legume and legume products were the second most consumed foods at breakfast, the number of consumers was still only ≈33%. In Malaysia, typical dishes consumed at breakfast include nasi lemak and nasi goring for Malays, roti canai and chapati for Indians, and noodle soup and fried rice among Chinese [23]. However, there appears to be considerable porosity in the choice of these breakfast foods across ethnic groups. These Asian breakfast foods are usually cooked composite dishes requiring skilled culinary techniques and are therefore largely prepared and purchased outside the home. The higher fat, sodium and sugar intakes at breakfast noted in Malaysia may be related to these traditional dishes. Previous research conducted in Malaysia has indicated that sauces, which are commonly found in the dishes, play a significant role in contributing to sodium intake [24]. Furthermore, the Malaysian Food Barometer reported that Western-style foods, such as bread, biscuits/croissants, and sandwiches, were consumed by about 9% of the population, and this could also be a contributory factor to higher intakes of sodium, sugar, and fat.

Another notable finding was that Malaysians tended to have the highest intakes of most micronutrients at breakfast, particularly vitamin B, iron, magnesium, and potassium. This might be due to a higher animal protein availability in Malaysia compared to Indonesia and the Philippines [25]. It is noteworthy that in Indonesia, wheat flour, cooking oil and refined sugar are fortified with iron, zinc, folic acid, and vitamins B1 and B2, and in the Philippines, rice and wheat flour are fortified with iron [26]. However, Malaysia does not have mandatory fortification practices of this nature [26].

Vitamin A intake at breakfast was noticeably higher in Indonesia, while vitamin D intake was the lowest among the three countries. Previous studies in Indonesia have shown variability in reported vitamin A intakes which may be related to the dietary recall method [27]. The one-day recall used in the present study may not be reliable in estimating usual vitamin A intake, so these results should be interpreted with caution. Furthermore, mandatory vitamin A fortification of some staple foods is in place in Malaysia (condensed milk and margarines) and the Philippines (wheat flour and sugar) but not in Indonesia [26]. Low vitamin D intake has previously been reported in Indonesia [28] and was confirmed by reports of low serum 25 (OH) D levels [27]. Mandatory fortification programs with vitamin D do not exist in any of the countries [29], so the variation may relate to differences in voluntary fortification practices or intake of natural food sources such as fish and eggs. The overall low intake of fibre and vitamin C in all countries likely relates to a low consumption of fruit and whole grains at breakfast. Notably, fruit was not among the top 10 foods consumed at breakfast in the Philippines and in Indonesia, with only 3% of the study population reporting consumption of fruit and fruit juices at breakfast [7,9]. The limited data available on whole-grain consumption in the Philippines and Malaysia indicate that intakes are very low [30].

Although breakfast made a significant contribution to daily intakes for many nutrients, as outlined in Figure 2, when the adequacy of daily intakes is taken into consideration (Table 2), the contribution of breakfast remained substantial for vitamin B, iron and calcium but diminished for several other nutrients, including fibre, vitamins C and D, zinc, and potassium. For example, breakfast provided on average 25–30% of daily fibre intakes but only 6–13% of the RDA for fibre. Therefore, it was important for us to consider these two elements when developing the guiding principles. Like other phases of the IBRI project, we assessed breakfast intakes according to tertiles of overall diet quality using the NRF 9.3 Index with the objective of using intakes in the upper tertiles as realistic targets. However, in contrast to other regions, we observed only very small differences for these nutrients across the tertiles, indicating an overall low intake across the population. Therefore, in developing guiding principles for optimal intakes of nutrients that are reasonably attainable across all three countries, a degree of flexibility was needed, and subjective judgement played some role in deciding the recommendations.

The proposed recommendations have the potential to empower countries with an evidence base to enhance their current food-based guidelines for breakfast, and, if not already established, to develop guidelines that specifically address the highlighted nutrients of concern. Furthermore, they can help to underpin educational campaigns and programs on breakfast. The breakfast recommendation targets we propose generally align with the dietary guidelines of Indonesia, which suggest that breakfast should account for 15–30% of daily nutrient needs. However, we propose a minimum of 20% of daily intake for energy, based on observed intakes during breakfast. Additionally, we have provided more specific targets for each nutrient, rather than a generalised target range. The methodology utilised in this study, as well as in all the IBRI studies, can provide guidance for other countries that have access to high-quality dietary data at the daily and breakfast intake levels. The targets proposed can be further tailored to specific target populations by using relevant national dietary reference values. Additionally, these recommendations can be valuable for countries in the South-East Asian region with comparable dietary patterns but limited resources to conduct their own population-based dietary surveys.

However, there are several limitations of the current study that should be mentioned. Firstly, it is evident that the sample size for the Philippines survey was magnitudes larger than those for the Malaysian and Indonesian surveys, and it is therefore more likely to be representative. In Indonesia, the survey was representative for around 50% of the population, but the population in the eastern part of Indonesia (e.g., Papua, Maluku, and Nusa Tenggara) was less represented. Furthermore, it is important to note that the dietary intake methodology varied among the countries. The Philippines employed a 2-day 24-h recall method, which allows for more accurate estimation of usual dietary intakes. On the other hand, Indonesia and Malaysia used a 1-day recall, which may have resulted in under- or overestimation of certain nutrients. Another limitation is the use of the Nutrient Rich Food (NRF) index to assess diet quality, as it was originally developed and validated in Western populations. However, in this study, national dietary reference values were incorporated into the NRF calculations. Lastly, it is worth mentioning that the study did not include Filipino adults aged over 60 years, which may limit the generalizability of the findings to this specific age group.

As a next step, further assessment of food intake at breakfast is needed, especially for Malaysia, to help understand key food contributions to nutrient intakes. This is needed to translate these nutrient recommendations into culturally appropriate food-based guidelines for breakfast. The recommendations could also be complimented by recommendations for children—an important target audience for breakfast education. Data for breakfast intake in Filipino children have already been published [9], so a national-level recommendation would be readily achievable. Another future progression could be the translation of the recommendations into a single breakfast quality score. This has already been completed and validated for the European and North American IBRI recommendations [31] and can serve as a useful tool to evaluate breakfast quality in populations and/or support education on breakfast.

## 5. Conclusions

In conclusion, the IBRI project is one of the first studies to have provided detailed quantitative data on breakfast nutrient intakes among adults in three South-East Asian countries and has done so using a harmonised methodology. Furthermore, it has proposed a logical decision tree to translate these data into targets for optimal breakfast intakes which can assist national nutrition policy makers to underpin breakfast provision programs or food-based dietary guidelines for the breakfast meal.

## Figures and Tables

**Table 1:** Socio-demographic characteristics and BMI status of breakfast consumers across the three countries.

	Malaysia	Indonesia *	Philippines *
Total sample (*n*)	1604	1333	32,255
Breakfast consumers (*n*)	1428 (89%)	1263 (95%)	31,091 (96%)
Age			
18–29 years	500 (35%)	270 (21.4%)	7272 **
30–59 years	816 (57.2%)	739 (58.5%)	23,819
≥60 years	112 (7.8%)	155 (12.3%)	N/A
Gender			
Male	664 (46.5%)	654 (51.8%)	14,886 (47.9%)
Female	764 (53.5%)	609 (49.2%)	16,205 (52.1%)
Urbanisation			
Rural	1302 (91.2%)	818 (64.7%)	20,105 (64.7%)
Urban	126 (8.8%)	446 (35.3%)	10,986 (35.3%)
Education Level			
Primary school or lower	122 (8.5%)	573 (45.4%)	8429 (27.1%)
Secondary school	879 (61.6%)	541 (42.8%)	12,607 (40.6%)
College/university	427 (29.9%)	148 (11.7%)	10,053 (32.3%)
BMI status			
(WHO Classification)			
Underweight	136 (9.5%)	118 (9.3%)	2038 (6.6%)
Normal weight	792 (55.5%)	685 (54.2%)	16,446 (52.9%)
Overweight	345 (24.2%)	233 (18.4%)	8320 (26.8%)
Obese	155 (10.8%)	225 (17.8%)	2534 (8.2%)

* Subject data missing for age, urbanisation, education level and BMI status in Indonesia and for education level in the Philippines ** Data for the Philippines are for participants aged 19–29 years.

**Table 2:** Nutrient intakes at breakfast for all breakfast consumers across the three countries.

	Malaysia	Indonesia	Philippines
Nutrients	Means + SDs	Medians + IQRs	Means + SEs
Energy (kcal)	474 ± 302	396 (294, 571)	426 ± 1.9
Carbohydrate (g)	62.0 ± 38.4	50.9 (37.8, 75)	76.9 ± 0.3
Sugar (g)	17.0 ± 13.2	1.9 (0.5, 9.4)	10.9 ± 0.1
Total fat (g)	18.4 ± 16.0	13.3 (7.6, 23.4)	7.4 ± 0.1
Saturated fat (g)	7.5 ± 7.2	5.1 (2.4, 10.2)	3.1 ± 0.03
Protein (g)	16.0 ± 12.4	12.8 (7.9, 20.5)	12.9 ± 0.1
Fibre (g)	3.2 ± 3.5	1.5 (0.5, 3.0)	1.9 ± 0.01
Vitamin A (mcg)	113.0 ± 116.7	156.4 (59.2, 356.8)	92.7 ± 3.5
Vitamin D (mcg)	0.6 ± 1.0	0.1 (0. 0.9)	0.8 ± 0.01
Vitamin C (mg)	6.6 ± 14.38	1.0 (0, 5.2)	3.5 ± 0.1
Thiamine (mg)	0.3 ± 0.2	0.2 (0.1, 0.3)	0.2 ± 0.002
Riboflavin (mg)	0.3 ± 0.3	0.2 (0.1, 0.4)	0.2 ± 0.002
Niacin (mg)	5.6 ± 4.6	3.2 (1.3, 5.6)	4.5 ± 0.02
Calcium (mg)	134.3 ± 106.0	120.2 (65.9, 222)	80.0 ± 0.8
Iron (mg)	3.8 ± 3.6	2.7 (1.7, 5.0)	2.1 ± 0.01
Potassium (mg)	692.4 ± 393.1	244.6 (93.3, 467.6)	317.6 ± 1.6
Magnesium (mg)	65.8 ± 44.8	30.0 (15.3, 59.2)	38.8 ± 0.2
Zinc (mg)	1.7 ± 1.5	1.3 (0.7, 2.7)	1.4 ± 0.1
Sodium (mg)	545.8 ± 784.3	285.7 (114.2, 593.1)	261.1 ± 3.2

**Table 3:** Codex NRVs and nutrient intakes (expressed as %s of Codex NRVs) for the whole day, at breakfast for all breakfast consumers and at breakfast in the upper tertile of the NRF 9.3 Index across the three countries: Malaysia (MY), Indonesia (ID) and the Philippines (PH).

Nutrient *	Codex NRVs	Daily Intake (% of Codex NRV)			Intake at Breakfast (% of Codex NRV)			Intake at Breakfast in Highest NRF Tertile (% of Codex NRV)		
		MY	ID	PH	MY	ID	PH	MY	ID	PH
Energy	2000 Kcal	90	81	90	24	20	21	24	20	25
Protein	50 g	150	115	120	32	26	27	32	30	36
Fibre	25 g	48	25	33	13	6	12	12	8	12
Vitamin A	800 μg	57	104	40	14	20	9	16	26	17
Vitamin	5 μg	44	36	64	12	2	16	14	2	30
Vitamin C	100 mg	84	13	40	7	1	1	7	3	8
Thiamine	1.2 mg	92	58	68	25	17	17	25	17	23
Riboflavin	1.6 mg	81	63	51	19	13	13	25	19	17
Niacin	18 mg	125	72	108	33	18	27	37	24	35
Calcium	800 mg	54	63	41	17	15	15	18	22	16
Magnesium	300 mg	79	48	59	23	10	14	26	18	21
Iron	14 mg	111	94	64	28	19	15	28	26	21
Zinc	11 mg	54	56	50	15	12	13	12	15	14
Potassium	3500 mg	66	30	37	18	7	9	20	9	13
Sodium	2000 mg	150	68	46	37	14	12	27	10	8

* Daily and breakfast nutrient intakes are expressed as averages for Malaysia and the Philippines and as medians for Indonesia.

**Table 4:** Proposed nutrient recommendations for breakfast for adults in the three South-East Asian countries.

Nutrient	Principle	WHO/Codex Daily Recommended Values *	Breakfast Recommendation (% of WHO/Codex Values) *	Breakfast Recommendation
Energy	1	-	-	400–500 kcal based on an average 2000 kcal diet or 20–25% of daily energy
Carbohydrate	2	55–75% daily energy	55–75% breakfast energy	
Protein	2	50 g	25–30%	12.5–15 g
Niacin	2	18 mg	≥25%	4.5 mg
Vitamin D	3	5 mcg	≥15%	0.75 mcg
Calcium	3	800 mg	≥20%	160 mg
Magnesium	3	300 mg	≥20%	60 mg
Iron	3	14 mg	≥25%	3.5 mg
Riboflavin	3	1.6 mg	≥20%	0.32 mg
Thiamin	3	1.2 mg	≥20%	0.24 mg
Vitamin A	3	800 mcg	≥20%	160 mcg
Sodium	3	2000 mg	Max 15%	<300 mg
Fibre	4	25 g	≥15%	3.8 g
Vitamin C	4	100 mg	10–15%	10–15 mg
Potassium	4	3500 mg	≥15%	525 mg
Zinc	4	11 mg	≥15%	1.7 mg
Free Sugars	5	<10% daily energy	<10% breakfast energy	
Total Fat	5	<30% daily energy	<30% breakfast energy	
Saturated Fat	5	<10% daily energy	<10% breakfast energy	

* Carbohydrate, free sugar, total fat, and saturated fat recommendations were guided by WHO recommendations, while all other nutrients except for energy are expressed as percentages of Codex NRVs.

## Data Availability

Publicly available data from the 2018 Expanded National Nutrition Survey by the Food and Nutrition Research Institute—Department of Science and Technology is available at http://enutrition.fnri.dost.gov.ph/ (accessed on 1 November 2022). The process of making publicly available the dataset from the 2018 Malaysian Food Barometer by the Chair of Food Studies “Food Cultures and Health” based on Open Science philosophy is in progress. Raw datasets are available on request from the corresponding author of the original manuscript.
